# Pulmonary function in non-hospitalized adults and children after mild COVID-19: a single-centre prospective cohort study

**DOI:** 10.1186/s12890-025-04007-y

**Published:** 2025-11-06

**Authors:** Alexandra R. Görges, Philipp Warnke, Micha Löbermann, Antonia Zapf, Julia Weitzel, Maya-L. Steinbach, Nadja Züfle-Lemke, Dagmar-C. Fischer, Manfred Ballmann

**Affiliations:** 1 Department of Pediatrics, Rostock University Medical Centre, Rostock, Germany; 2 Institute of Medical Microbiology, Virology, and Hygiene, Rostock University Medical Centre, Rostock, Germany; 3 Department of Tropical Medicine, Infectious Diseases and Nephrology, Rostock University Medical Center, Rostock, Germany; 4 Institute for Medical Biometry and Epidemiology, University Medical Center Hamburg-Eppendorf, Hamburg, Germany

**Keywords:** Mild COVID-19, Pulmonary function, Lung clearance index, Lung function testing, SARS-CoV-2

## Abstract

**Background:**

Although the majority of patients suffering from COVID-19 (coronavirus disease 2019) did not require hospitalization, data on the persistence of pulmonary sequelae after a mild SARS-CoV-2 (severe acute respiratory disease coronavirus 2) infection in patients without pre-existing respiratory diseases are virtually missing.

**Methods:**

Pulmonary function (spirometry, diffusion capacity for carbon monoxide (DLCO), and lung clearance index (LCI)) was assessed 4–12 weeks after SARS-CoV-2 infection in non-hospitalized patients aged 6–60 years. Acute and persisting COVID-19-related respiratory symptoms were ascertained, and the presence of other acute respiratory infection pathogens was checked via a multiplex-PCR approach from pharyngeal swabs. Participants with impaired lung function underwent a follow-up examination 3 months later. To complement the initial analysis, a retrospective z-score analysis of lung function parameters was conducted.

**Results:**

110 patients (90 adults, 20 children) were included. In 45 adults (50%) and 17 children (85%), at least one pulmonary function test indicated an impaired lung function, particularly the LCI. Despite overall improvement between baseline and follow-up, 9 of 13 (69.2%) children and 35 of 42 (83.3%) adults with initial impairment still showed abnormal values about 5 months post SARS-CoV-2 infection.

Specific respiratory symptoms were linked to lower spirometry and DLCO values. In 25 patients (40.3%) with abnormal lung function, and in 20 participants (43.5%) with normal pulmonary function, pathogens were detected at t_1_. The retrospective z-score analysis closely mirrored the initial findings.

**Conclusion:**

Critical pulmonary impairment after mild COVID-19 is rarely detected by spirometry and DLCO but may affect the LCI. Within 3 months, impaired pulmonary function improved in most patients. Children were less affected by severe pulmonary sequelae and respiratory complaints than adults. Complaints like dyspnoea or chest pain may be an early indicator of lung function impairment, suggesting that further diagnostic tests for treatable post-COVID-19 complications may be needed. Additionally, PCR-based screening for additional respiratory pathogens may help avoid misinterpretation of lung function results post SARS-CoV-2 infection.

**Trial registration:**

Retrospectively registered in ClinicalTrials.gov (ID NCT06318208) and German Clinical Trials Register (ID DRKS00030025).

**Supplementary Information:**

The online version contains supplementary material available at 10.1186/s12890-025-04007-y.

## Background

In December 2019, the first patients with SARS-CoV-2 (severe acute respiratory syndrome coronavirus 2) infections were documented, and the name COVID-19 (coronavirus disease 2019) was coined for the disease. SARS-CoV-2 is a positive-sense, single-stranded RNA virus, belongs to the family Coronaviridae and causes symptoms ranging from those typically seen in a mild cold to severe lung dysfunction and pneumonia [1]. Accordingly, COVID-19 is classified as mild, moderate, and severe. While in children and adolescents symptoms and duration of COVID-19 are generally mild, severe courses of the disease are preferentially seen in patients with higher age and/or those with concomitant chronic diseases [2, 3].

Symptoms that persist for more than 12 weeks or occur newly after the initial infection are summarized as long COVID or post-COVID syndrome [4, 5]. The prevalence of long COVID increases with severity of the acute infection, as well as in older patients and/or pre-existing pulmonary diseases, e.g., respiratory distress, asthma and/or a history of smoking [1, 6–10]. However, about 10–30% of non-hospitalized COVID-19 patients with initially mild SARS-CoV-2 infection suffer from shortness of breath and other symptoms of long COVID [11]. In some of these cases MRI- and/or CT-based imaging revealed pulmonary abnormalities independent of the severity of the initial infection [12–14]. Therefore, it appears reasonable to search for lung function impairment after mild COVID-19. Since the quantitative assessment of the lung clearance index (LCI) has been introduced as a sensitive, non-invasive technique for assessing lung function [15], this method seems well suited for screening in patients with persistent pulmonary symptoms after SARS-CoV-2 infection.

We report the results of a prospective, single-centre study designed to examine lung function in a large number of non-hospitalized children and adults at least 4 weeks after a mild, PCR-confirmed SARS-CoV-2 infection in the absence of known pre-existing respiratory diseases. To account for the impact of respiratory infections at time of lung function testing, all participants were screened (multiplex-PCR) accordingly.

## Patients and methods

### Study design and cohort

This prospective, single-centre study was approved by the local ethics committee (A 20212-0080) and registered in the German Clinical Trials Register (ID: DRKS00030025), as well as with ClinicalTrials.gov (ID: NCT06318208). Participants were enrolled between April 2021 and October 2021.

Records from all patients diagnosed as SARS-CoV-2-positive via PCR-based testing were retrieved from the clinical information system, and those aged between 6 and 60 years at the time of SARS-CoV-2-infection were contacted via phone call. At this time, patients were informed about the aim of the study and interviewed with respect to the inclusion criteria (mild course of disease, no hospitalization due to COVID-19) and exclusion criteria (pre-existing lung diseases, history of smoking within the last 5 years, SARS-CoV-2 vaccination, pneumonia within 8 weeks prior to SARS-CoV-2 infection). Those deemed eligible were invited to participate in the study at least 4 weeks after the PCR-confirmed diagnosis of COVID-19. Additionally, family members of volunteers tested in other local test centres were invited to participate after providing evidence of a SARS-CoV-2 infection. All participants and/or their legal guardian gave written informed consent. Examinations included a thorough assessment of pulmonary function, a pharyngeal swab for molecular detection of current respiratory infections, and a study-specific questionnaire on symptoms and duration of the acute SARS-CoV-2 infection, as well as on any persisting complaints (English version in Supplementary File 1). The analysis was limited to pulmonary symptoms, given their primary relevance to the study’s objective. Results of the lung function testing were handed over to the patients for recommended transfer to their family doctor or paediatrician.

To specifically assess changes and the persistence of lung function abnormalities following SARS-CoV-2 infection, only participants with at least one abnormal pulmonary function parameter at baseline were invited for a follow-up examination three months later. This allowed the study to focus on individuals at higher risk of persistent impairment and to make efficient use of diagnostic resources.

### Assessment of pulmonary function

Pulmonary function tests were performed according to ATS/ERS standards [16]. After a detailed explanation of the procedure, the participants were seated in an upright and relaxed position while breathing through a mouthpiece and wearing a nose clip. The EasyOne Pro^®^ system (ndd Medizintechnik AG, Zürich, Switzerland) was used for spirometry as well as for LCI 2.5% (nitrogen multiple breath washout, MBW) and DLCO (single breath washout with Helium and CO as tracer gases) [17]. DLCO results were corrected for hemoglobin. The lower limit of normal (LLN) percentage predicted for DLCO, forced vital capacity (FVC), and forced expiratory volume in 1 s (FEV_1_) were set at 80%, respectively [17–19]. For LCI, at least two technically acceptable measurements were required for the examination to be considered valid and values ≥ 7.0 in children and ≥ 7.5 in adults were defined as above the upper limit of normal (ULN) [15, 20, 21]. At follow-up, a change of ≥ 10% in % predicted values (spirometry, DLCO) or in absolute values (LCI) compared to baseline was considered clinically significant [19, 22–25].

In addition to the initial analysis, a retrospective z-score analysis was conducted using both the official GLI calculator and device-generated z-scores based on GLI reference values [18, 26–28]. A LLN of < −1.64 z-scores (spirometry, DLCO) and an ULN of >1.64 z-scores (LCI) were applied [22, 29]. A change of ≥ 1 z-score from the previous value was considered clinically significant [23].

### Analysis of pharyngeal swabs

Pharyngeal swabs were collected using Copan eSwabs^®^ with liquid Amies medium (Copan, Brescia, Italy) and stored at −80 °C until analysis. Samples were assessed applying the Seegene Allplex Respiratory Panels 1, 2, 3 and 4 (Seegene, Seoul, Korea), in combination with an automated liquid handling workstation (Seegene NIMBUS, Seoul, Korea) and the Bio-Rad CFX96 real-time PCR-Detection System (Bio-Rad Laboratories Inc., Hercules, California, USA). Results were evaluated utilizing the Seegene Viewer for real time instruments V3 software (Seegene, Seoul, Korea). Furthermore, the Aptima SARS-CoV-2/Influenza Assay (Hologic, San Diego, USA) was performed on the fully automated Hologic Panther System (Hologic, Massachusetts, USA).

### Statistics

The IBM SPSS Statistics^®^ (Statistical Package for Social Science), Version 27.0.1.0 was used, and participants were categorized according to age as children/adolescents (< 18 years) and adults (≥ 18 years). Normal distribution of data was checked both graphically and statistically for all lung function parameters. Continuous variables are given as mean ± standard deviation (for normally distributed data) and median with interquartile ranges (for non-normally distributed data). Categorical variables are reported as absolute numbers and percentages. Unpaired and paired tests were applied for comparison between groups (children vs. adults) or the time of examination ((t_1_) vs. (t_2_)). The Pearson and Spearman coefficients of correlation were calculated to investigate associations between normally and non-normally distributed variables. Where appropriate (especially in cases of small sample sizes or non-normal distribution), non-parametric tests such as Mann-Whitney-U test and Wilcoxon signed-rank test were applied.

## Results

### Characteristics of the study population

**Fig. 1:**
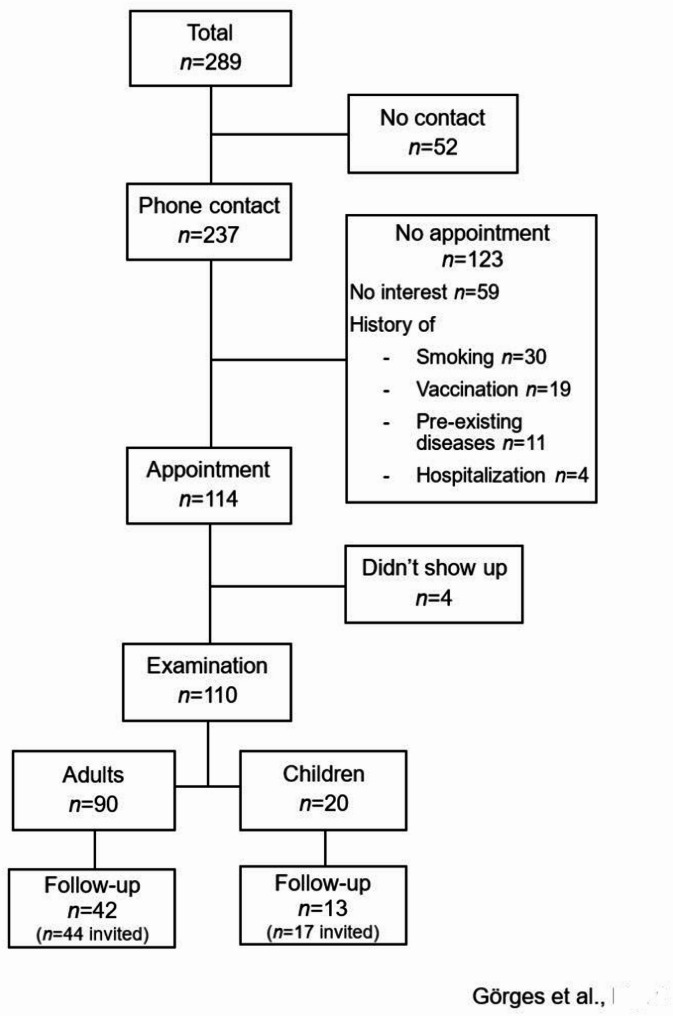
Flow chart of the patients included in the study. *n*: absolute number of patients

Overall, 289 patients were deemed eligible, and roughly half of them fulfilled the inclusion criteria (Fig. 1).

Finally, 90 adults (47 male) and 20 children (9 male) consented to participate, and examinations were performed at a median of 7.9 (4.3–11.3) weeks (t_1_) after the PCR-based diagnosis of SARS-CoV-2. The interval between t_1_ and the follow-up examination (t_2_) was 12.3 (11.0–16.7.0.7) weeks. The anthropometric characteristics of the study population as well as the results of the lung function assessments are presented in Table 1.

**Table 1:** Anthropometric data and results of lung function testing

		Children *n* = 20			Adults *n* = 100	
	t_1_	with follow-up (t_1_)	t_2_	t_1_	with follow-up (t_1_)	t_2_
Patients, *n*	9 m/11f	5 m/8f	5 m/8f	47 m/43f	26 m/16f	26 m/16f
Age [y]	12.7 ± 3.3	11.7 ± 3.4	11.9 ± 3.3	37.1 ± 12.7	39.2 ± 12.8	39.4 ± 12.8
BMI [kg/m^2^]	19.2 ± 2.4	19.5 ± 2.7	19.4 ± 2.7	25.6 ± 5.5	26.6 ± 6.5	26.7 ± 6.5
FEV1						
% of predicted	88.3 ± 14.1	89.4 ± 16.5	96.9 ± 11.9	95.4 ± 11.5	92.6 ± 11.4	93.6 ± 9.6
z-score	−1.00 ± 1.20	−0.89 ± 1.40	−0.26 ± 1.02	−0.38 ± 0.90	−0.85 ± 0.91	−0.52 ± 0.79
FVC						
% of predicted	87.6 ± 14.6	89.0 ± 16.0	98.2 ± 12.9	92.8 ± 11.4	89.4 ± 12.1	90.4 ± 11.0
z-score	−1.06 ± 1.23	−0.93 ± 1.35	−0.16 ± 1.07	−0.58 ± 0.92	−0.85 ± 0.97	−0.77 ± 0.90
DLCO				(*n* = 87)	(*n* = 41)	
% of predicted	89.2 ± 14.6	89.5 ± 18.2	91.2 ± 14.9	106.6 ± 12.9	104.2 ± 12.1	103.4 ± 12.3
z-score	−0.52 ± 0.71	−0.50 ± 0.88	−0.43 ± 0.73	0.48 ± 0.95	0.30 ± 0.88	0.25 ± 0.88
LCI						
Absolute	7.80 ± 1.01	8.02 ± 1.10	7.80 ± 0.94	7.56 ± 1.10	8.35 ± 0.98	8.04 ± 1.07
z-score	2.78 ± 1.38	2.97 ± 1.45	2.76 ± 1.65	2.15 ± 2.01	3.30 ± 1.23	2.80 ± 2.92
						Görges et al. Tbl. 1

Continuous variables are expressed as mean value ± standard deviation

*BMI* Body Mass Index, *FEV*_1_ Forced Expiratory Volume in 1 s, *FVC* Forced Vital Capacity, *DLCO* Diffusion Capacity for Carbon Monoxide, *LCI* Lung Clearance Index, *n*: absolute number of patients (*m* male, *f* female)

### Assessment of pulmonary function

At the time of the first examination, 45 adults (50%) and 17 children (85%) had at least one abnormal value in pulmonary function tests according to predefined criteria. In particular, FEV_1_ was below normal in 9 adults (10%) and 4 children (20%), FVC was below normal in 10 adults (11.1%) and 6 children (30%), while a reduced DLCO was detected in 5 children (25%) but in none of the adults. In contrast, an impaired LCI was present in 15 children (75%) and 38 adults (42.2%). Furthermore, in 6 adults (6.7%) as well as in 5 children (25%), abnormal LCI results were accompanied by a reduced FEV_1_ and/or FVC, whereas in 2 children (10%), spirometry results, DLCO, and LCI were out of normal range. No apparent correlation was observed between the duration from positive SARS-CoV-2 multiplex-PCR to the first assessment and lung function results.

Despite an overall improvement of lung function between baseline and follow-up, 9 of 13 (69.2%) children and 35 of 42 (83.3%) adults with impaired pulmonary function at baseline still had at least one abnormal value at the follow-up examination roughly 5 months after SARS-CoV-2 infection.

**Fig. 2:**
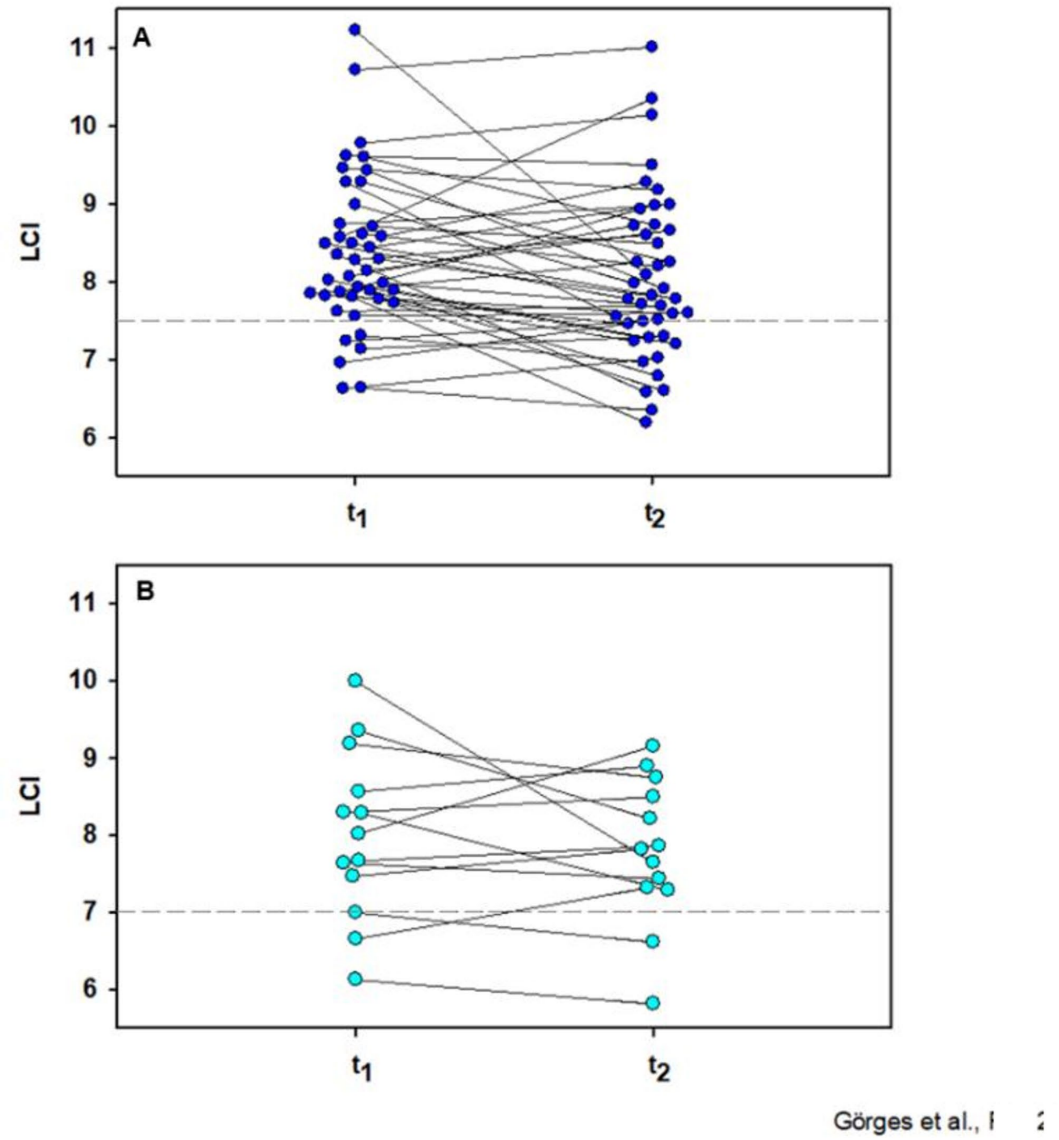
Changes in absolute lung clearance Index between t_1_ and t_2_ in adult (**A**) and pediatric (**B**) patients. The upper limit of normal is indicated with a dashed line, A: *n* = 42, B: *n* = 13

General improvements in initially abnormal lung function parameters were observed in 9 of 13 patients (69.2%) with abnormal FVC, in 4 of 5 children (80%) with abnormal DLCO, and in 29 of 47 patients (61.7%) with previously impaired LCI (Fig. 2).

Even when considering only participants with initially abnormal values for each lung function parameter, improvements were observed in FEV_1_ (*n* = 10, t_1_(77.0 ± 6.0) vs. t_2_(83.0 ± 13.0), Z = 2.81, *p* = 0.005), FVC (*n* = 13, t_1_(72.0 ± 6.0) vs. t_2_(76.0 ± 8.0), Z = 2.45, *p* = 0.014), and LCI (*n* = 47, t_1_(8.30 ± 1.32) vs. t_2_(7.98 ± 1.20), Z=−2.25, *p* = 0.015), as determined by Wilcoxon signed-rank tests. Only DLCO showed no notable change between the first and second assessments.

Clinically significant improvements (≥ 10%) in adult participants were seen in FVC (1 of 10), FEV_1_ (2 of 8), and LCI (10 of 36), with 2 adults showing LCI deterioration.

In children, improvements ≥ 10% occurred in FVC (2 of 3), in FEV_1_ (2 of 2), and DLCO (2 of 5), while one child worsened in DLCO. LCI showed clinically significant improvement in 3 of 11 (27.3%) children, while one child had a deterioration.

### Evaluation of pulmonary symptoms during the SARS-CoV-2 infection and at time of the study examinations

**Fig. 3:**
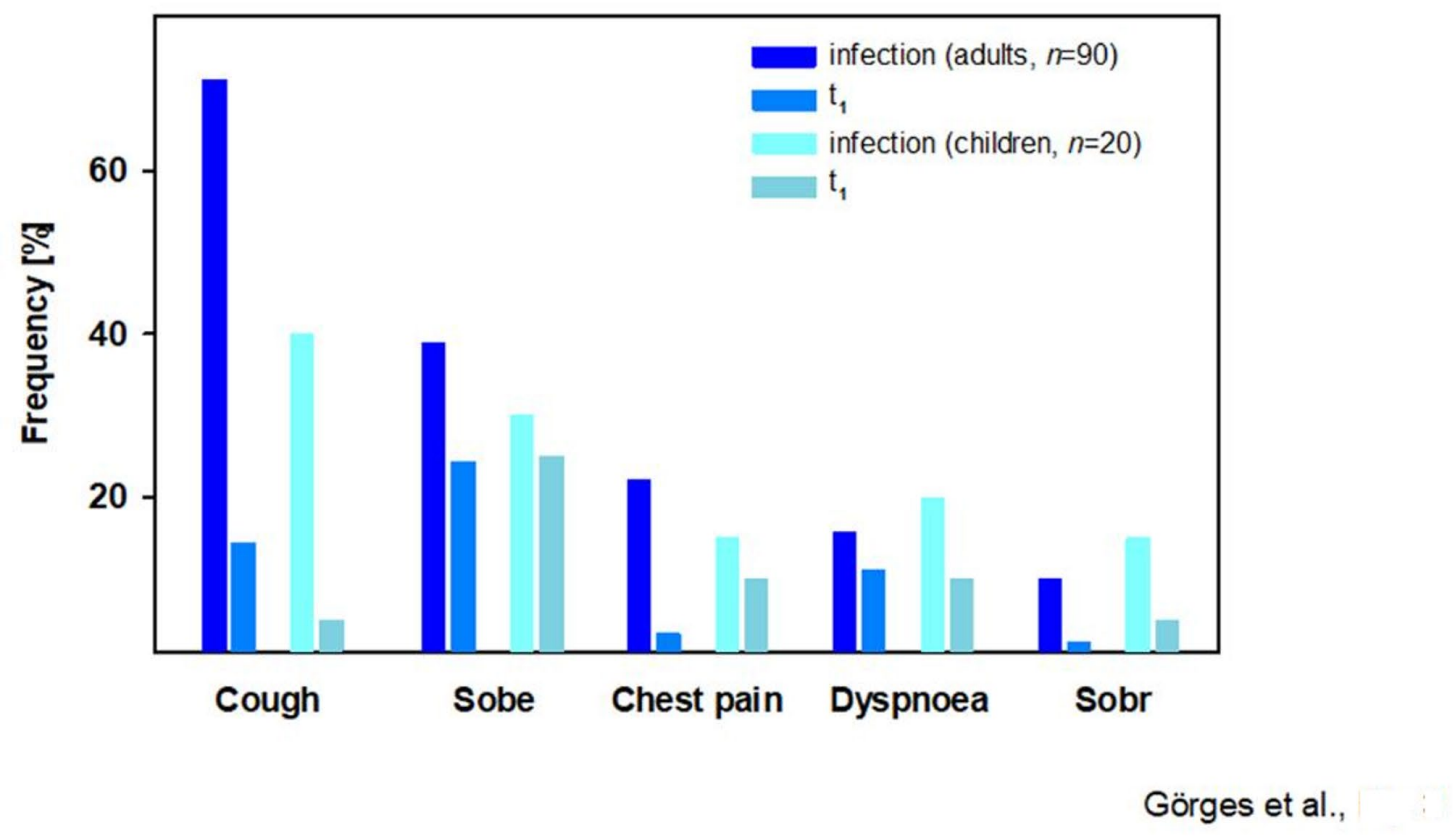
Symptoms during acute SARS-CoV-2 infection and at time of enrolment in adult and pediatric patients. Sobe: shortness of breath during exertion, sobr: shortness of breath at rest

The frequency of pulmonary symptoms during SARS-CoV-2 infection and at the time of the examinations was markedly higher in adults compared to children (Fig. 3). However, the average number of symptoms during COVID-19 was higher in younger patients. 20 adults (22.2%) and 6 children (30%) reported 3 to 5 respiratory symptoms during the acute phase of infection. Most patients (37.3%) stated a duration of 8 to 14 days of the acute symptomatic SARS-CoV-2 infection, while 26.4% reported a course of less than 8 days, and 21.8% a duration of more than 14 days. At the time of the follow-up examination, 1 of 13 (7.7%) children and 12 of 42 (28.6%) adults reported persisting shortness of breath during exertion, and 2 adults (4.8%) had more than one lasting complaint.

**Fig. 4:**
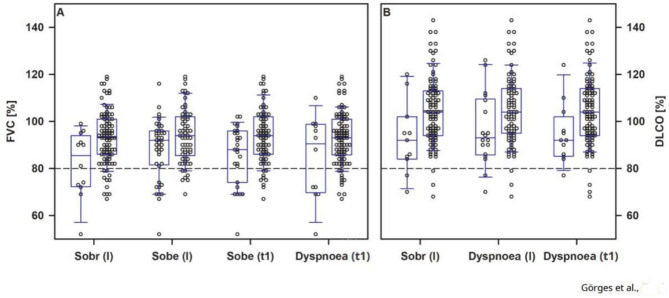
FVC [%] (**A**) and DLCO [%] (**B**) comparing patients with and without specific respiratory symptoms. I: during acute SARS-CoV-2 infection. **A** *n* = 110; Sobr: shortness of breath at rest (with: *n* = 12 vs. without: *n* = 98), Sobe: shortness of breath during exertion (I: with: *n* = 41 vs. without: *n* = 69; t_1_: with: *n* = 27 vs. without: *n* = 83), Dyspnoea (with: *n* = 12 vs. without: *n* = 98). **B** *n* = 107; Sobr (with: *n* = 11 vs. without: *n* = 96), Dyspnoea (I: with: *n* = 18 vs. without: *n* = 89; t_1_: with: *n* = 12 vs. without: *n* = 95)

Within the whole cohort, a moderate correlation between age at onset of the disease and median duration of symptoms was observed (ρ = 0.44, *p* ≤ 0.001). Overall, 64.5% of participants with abnormal, and 84.8% with normal pulmonary function reported respiratory symptoms during COVID-19. Specific symptoms were linked to lower mean values in spirometry and DLCO during the initial examination. However, these findings did not correlate with an impaired LCI (Fig. 4A, B).

In adults, persistence of symptoms during the initial course of the SARS-CoV-2 infection was related to a lower DLCO (ρ=−0.36, *p* < 0.001) and a higher LCI (ρ = 0.27, *p* = 0.011) at t_1_. At the follow-up examination, differences in mean LCI were shown between 9 adults with chest pain (8.66 ± 1.57) and 33 adults without (7.87 ± 0.84, *p* = 0.048) chest pain during the acute infection.

In children, the age of those reporting pulmonary symptoms (*n* = 8, median 16.0 ± 5.0 years) during SARS-CoV-2 infection was considerably higher compared to those (*n* = 12, 11.5 ± 6.0 years) without symptoms (U = 76.5, Z = 2.22, *p* = 0.025). Children who experienced at least one respiratory symptom during the acute phase of infection demonstrated a lower DLCO at t_2_ (*n* = 8, median 85.0 ± 17.0) compared to asymptomatic children (*n* = 12, 99.0 ± 22.0; U = 6.0, Z=−2.05, *p* = 0.045).

### Results of multiplex-PCR testing

**Fig. 5:**
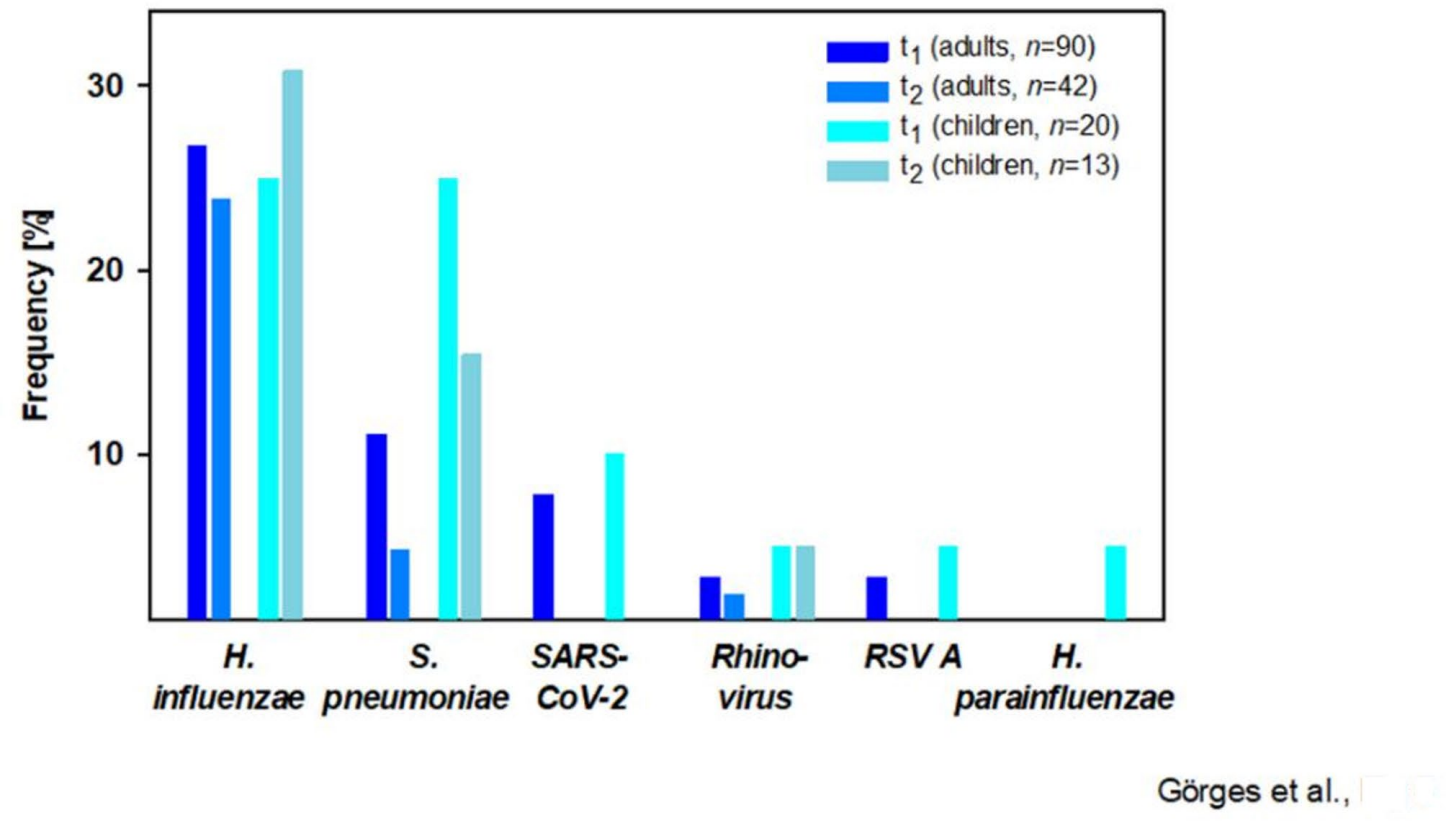
Overview of Multiplex-PCR results H. influenzae: *Haemophilus influenzae*, S. pneumoniae: *Streptococcus pneumoniae*, SARS-CoV-2: Severe Acute Respiratory Syndrome Coronavirus 2, RSV A: Respiratory Syncytial Virus A, H. parainfluenzae: *Haemophilus parainfluenzae*

Multiplex-PCR-based analysis of the pharyngeal swabs taken at t_1_ revealed a negative result in 65 (59.1%) patients, while at least one pathogen was identified in 34 adults (37.8%) and 11 children (55%), respectively (Fig. 5).

At the time of the follow-up, at least one pathogen was detected in pharyngeal swabs of 28.6% of adults (12 of 42) and 53.9% of children (7 of 13).

In 25 patients (40.3%) with abnormal lung function, and in 20 participants (43.5%) with normal pulmonary function, pathogens were detected at t_1_. Persistent cough was associated with the detection of rhinovirus (Fisher’s Exact Test, *p* = 0.006, φ = 0.36) and *Haemophilus influenzae* (χ^2^ [1] = 7.83, *p* = 0.005, φ = 0.27). A lower DLCO was noted in patients with a positive result for *Streptococcus pneumoniae* (91.9 ± 12.0 (*n* = 14) vs. 105.1 ± 14.5 (*n* = 93); *p* = 0.002).

At follow-up, LCI in children with a positive PCR result tended to be higher compared to those without detection of a pathogen (8.16 ± 0.60 (*n* = 7) vs. 7.37 ± 1.13 (*n* = 6), *p* = 0.135), whereas no such differences were noted in adults. Participants with a positive PCR result at t_2_ were considerably younger (*n* = 19, median 21.0 ± 29.0) compared to those without evidence of recurrent respiratory infection (*n* = 36, 40.0 ± 27.0; U = 175.5, Z=−2.95, *p* = 0.003).

### Retrospective analysis of lung function z-scores

Apart from minor differences, the z-score analysis showed results that were consistent with those of the initial analysis based on absolute and % predicted values.

**Fig. 6:**
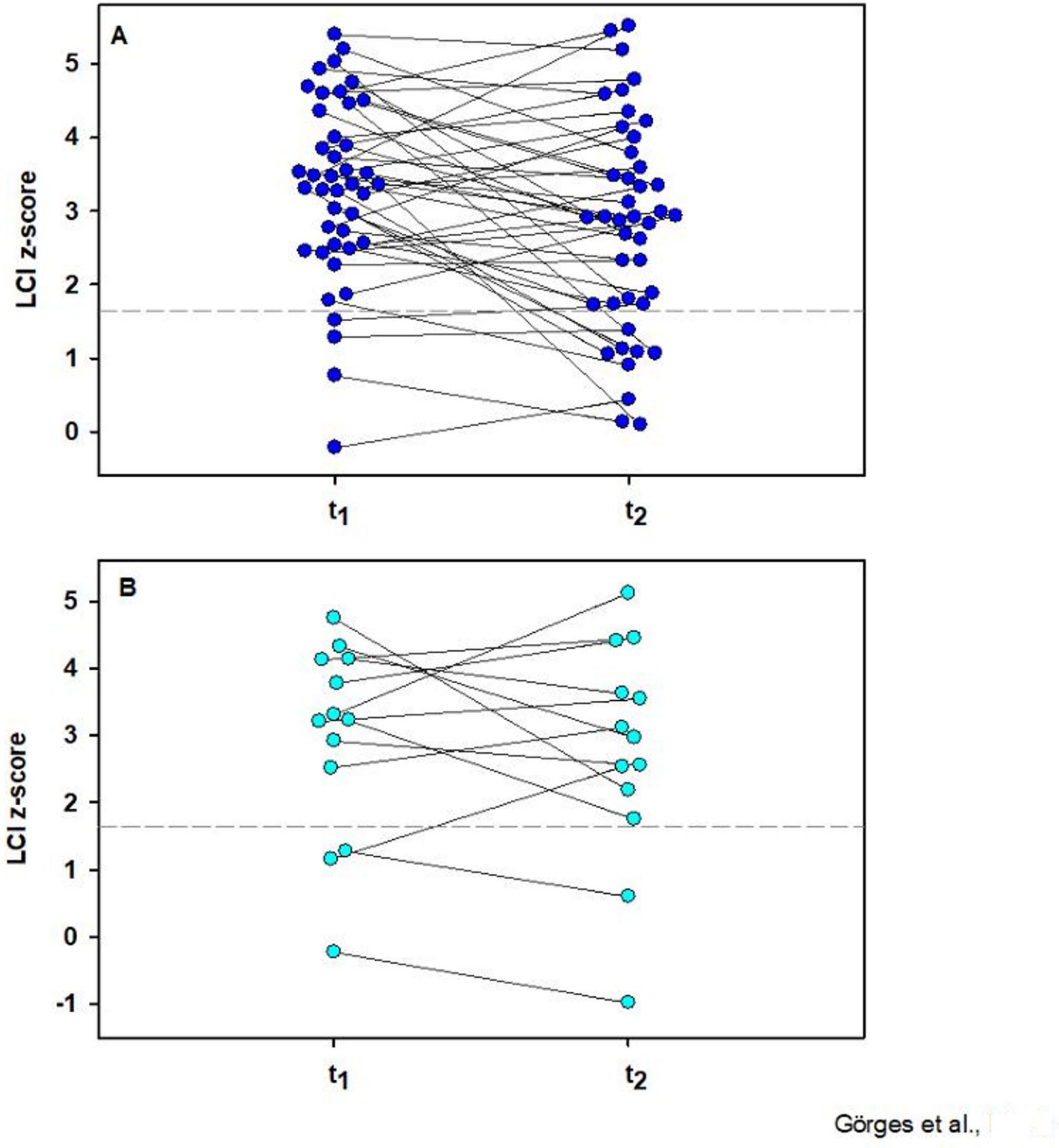
Changes in lung clearance index z-scores between t_1_ and t_2_ in adult (**A**) and pediatric (**B**) patients. The upper limit of normal is indicated with a dashed line, A: *n* = 42, B: *n* = 13

Spirometry impairments were identified in 20 patients (FVC: *n* = 16 (9.7%), including 6 children; FEV_1_: *n* = 13 (7.9%), including 4 children), whereas DLCO z-scores remained within normal limits in all participants. A total of 69 participants (62.7%, including 14 children) showed abnormal LCI z-scores. At the follow-up examination, an improvement exceeding 1 z-score was observed in 3 of 13 patients (1 adult, 2 children) with initially abnormal FVC and in 3 of 9 participants (2 adults, 1 child) with initially abnormal FEV_1_. A clinically significant improvement in previously abnormal LCI was observed in 15 of 48 patients (12 adults, 3 children), while a deterioration was noted in 4 patients (3 adults, 1 child). The remaining participants showed no clinically meaningful changes (Fig. 6A, B).

Among patients with previously impaired spirometry z-scores, both FEV_1_ (*n* = 9; t_1_: −1.93 ± 0.60 vs. t_2_: −1.23 ± 1.21; Z = 2.66, *p* = 0.008) and FVC (*n* = 13; t_1_: −2.14 ± 0.65 vs. t_2_: −1.89 ± 0.58; Z = 2.45, *p* = 0.014) improved, as determined by Wilcoxon signed-rank tests. In addition, LCI z-scores decreased in patients with previously abnormal values (paired t-test, *n* = 48; t_1_: 3.58 ± 0.90 vs. t_2_: 3.08 ± 1.29, *p* = 0.010).

Despite overall improvements in lung function between baseline and follow-up, abnormal z-scores were still present in 11 of 55 patients (20%) for spirometry and in 44 of 55 patients (80%) for LCI at the follow-up assessment approximately 5 months after SARS-CoV-2 infection.

No relevant associations were found between the persistence of respiratory symptoms and LCI z-scores. However, lower DLCO z-scores in adults were related to longer durations of respiratory symptoms, as demonstrated in previous analyses. Abnormal spirometry at baseline was associated with shortness of breath at rest during SARS-CoV-2 infection (Fisher’s Exact Test, *p* = 0.035, φ 0.23), persisting shortness of breath at rest (*p* = 0.036, φ = 0.28), and persisting dyspnoea at t_1_ (χ^2^ [1] = 7.97, *p* = 0.005, φ = 0.27). DLCO also tended to be lower in participants reporting dyspnoea compared to those without (*n* = 12: −0.31 ± 0.84 vs. *n* = 98: 0.37 ± 0.98, *p* = 0.023), though these findings were not associated with impaired LCI. Furthermore, neither persisting nor newly emerging symptoms at follow-up were associated with any deterioration in lung function parameters.

Correlations observed in the multiplex-PCR analysis using z-scores were consistent with those based on absolute and % predicted values, except that no association between impaired LCI and pathogen detection was found in children. Moreover, changes in spirometry, DLCO, and LCI between baseline and follow-up did not correlate with newly detected pathogens or changes in multiplex-PCR results from positive to negative.

## Discussion

About 90% of COVID-19 survivors experienced a mild course of the disease, and most patients suffering from long-COVID had not been hospitalized for SARS-CoV-2 infection [30]. In children and adolescents with SARS-CoV-2 infections, hospitalization rates are below 2%, and severe respiratory complications are rarely seen [3, 9, 31, 32].

Our data show that impaired pulmonary function and persisting respiratory complaints may be present even in otherwise healthy adults and children experiencing a mild acute SARS-CoV-2 infection. We considered the current respiratory infection status at the time of the study examinations as an additional confounder and excluded patients with pre-existing pulmonary diseases and/or a history of smoking during five years prior to the SARS-CoV-2 infection.

### Assessment of pulmonary function

Our data indicate that in the majority of patients suffering from a mild SARS-CoV-2 infection, pulmonary function impairment is detectable especially through LCI even after 7 to 8 weeks. Although lung function improved in more than half of them at the follow-up examination, normal values were not always reached.

In hospitalized adults with severe COVID-19, a pulmonary function impairment can persist for months, and DLCO was shown to decrease as severity of the infection and age go up [3, 33–40]. However, we excluded patients experiencing a SARS-CoV-2 infection who were older than 60 years or reported a history of pulmonary disease. Thus, it appears reasonable that the DLCO, particularly when expressed as z-scores, remained within normal limits in our patients. Moreover, structural interstitial changes like fibrosis, which reduce the DLCO, are not to be expected in younger patients with a mild SARS-CoV-2 infection [41].

Initially, most children showed elevated LCI, while spirometry and DLCO resembled the findings of previous studies in children [8, 42–44]. In contrast to our results, Knoke et al. detected abnormal LCI in only 4.4% (*n* = 3/68) and DLCO in 5.3% (*n* = 2/38) of children after mild COVID-19, with no relevant difference in frequency of impaired lung function between children with and without SARS-CoV-2 infection (16.4% vs. 27.7%) [3]. This might be due to *(i)* the interval between infection and assessment of pulmonary function and/or *(ii)* the utilization of different equipment. Moreover, it might well be that a thus far clinically silent and thus not yet diagnosed acute or chronic respiratory disease can cause an elevated LCI as well [39]. Since the LCI reflects ventilation efficiency, particularly in the peripheral small airways, accumulation of mucus and/or inflammation within these peripheral airways is likely to affect ventilation homogeneity even after mild SARS-Cov-2 infection [20, 40]. In fact, in previous studies, abnormal lung perfusion and air trapping were detected through imaging studies in non-hospitalized patients, as well as pulmonary fibrosis in chest X-rays in some children one month after SARS-CoV-2 infection [11, 43].

In summary, the LCI turned out to be a highly sensitive indicator of peripheral lung function impairment in our patient group. Although in the majority of our patients, normal values were not reached within the 5-month observation period, our results indicate an overall improvement of lung function. This is well in line with a recent report on pulmonary restriction even 6 months after hospitalisation because of COVID-19 [45].

Although severe functional and structural pulmonary damage after mild SARS-CoV-2 infection is rarely to be expected in children, it appears reasonable to include LCI in pulmonary function testing in these COVID-19 patients to detect early pulmonary changes [15], given that both spirometry and DLCO revealed no or only minor abnormalities.

### Respiratory symptoms

In line with the current literature, the majority of children in our cohort were free of respiratory symptoms during acute SARS-CoV-2 infection [2, 3, 43, 46]. Nevertheless, one third of our participants reported common pulmonary symptoms lasting 7–8 weeks, while symptoms persisting for more than 5 months were rarely seen and probably associated with pulmonary inflammation [33, 47–53].

Our study revealed no considerable differences in pulmonary function, especially regarding LCI, between SARS-CoV-2-infected patients with and without respiratory symptoms. As stated by previous studies, a partial mismatch between the results of lung function tests and pulmonary complaints at the time of examination was observed [3, 34, 54]. Normal lung function with lasting pulmonary symptoms may indicate dysfunctional breathing or new underlying symptomatic respiratory infections [3]. However, the mechanisms behind pulmonary function impairment and respiratory symptoms due to COVID-19 may not be identical. Supporting the findings of a Mexican study, lower spirometry and DLCO results, as well as higher mean LCI, were associated with a longer duration of initial symptomatic infection and/or specific symptoms like persisting dyspnoea [55]. As LCI remained abnormal largely independent of respiratory symptoms, whereas spirometry and DLCO correlated with specific symptoms such as persisting shortness of breath, it is possible that the LCI may be more sensitive to subclinical pulmonary changes. Nevertheless, specific respiratory symptoms may serve as early indicators of pulmonary function impairment.

### PCR-based screening for respiratory pathogens

The impact of pathogen detection on lung function was low and only visible in some patients. As expected, the prevalence of pathogens was higher in children compared to adults. No relevant correlations of persisting symptoms after COVID-19 and a positive PCR result were detected, except for lasting cough with rhinovirus and *Haemophilus influenzae*. Therefore, some persisting symptoms after SARS-CoV-2 infection may be attributable to newly acquired infections, not due to COVID-19. Supporting this theory, one study demonstrated that 31.1% of participating children had a symptomatic infection other than SARS-CoV-2 within the last 6 months. In contrast to our study, this was anamnestic data and not proven by PCR or respiratory tract culture [3].

The proportion of patients with and without reduced pulmonary function was nearly the same among those with positive and negative PCR outcome. Normal lung function despite a pathogen detection may be explained by bacterial or viral colonization or underlying infection without detectable pulmonary impairment. However, respiratory tract infections can decrease lung function parameters, e.g. by a decrease in DLCO in the context of detection of *S. pneumoniae* [3, 13, 56].

Therefore, it appears feasible to complement assessment of pulmonary function after SARS-CoV-2 infection with a PCR-based screening for other respiratory pathogens that may cause additional pulmonary sequelae, particularly in cases of persistent or newly emerging respiratory symptoms.

## Conclusion

Critical pulmonary impairment after mild COVID-19 is rarely detected by conventional spirometry and DLCO, but mild impairment due to infection may be revealed by LCI. Only a minority of patients develop symptoms of long-COVID after an initially mild infection, and children are even less frequently affected by persisting respiratory complaints and severe pulmonary impairment compared to adults. Nevertheless, children and adults with lasting COVID-19 symptoms and/or persisting or worsening pulmonary sequelae should be thoroughly monitored, and further diagnostics may be initiated. Furthermore, we support the inclusion of multiplex-PCR in lung function testing to better understand the influence of respiratory pathogens on lung function.

### Limitations

All data regarding pre-existing diseases and smoking history are based on patients’ statements and were not confirmed by pre-existing data (e.g., pulmonary function tests). Some participants may have had prior experience with lung function testing, which could have influenced their performance due to increased procedural familiarity. In the absence of prior pulmonary function test results, no comparative pre-infection data could be established. Symptoms during SARS-CoV-2 infection and persistent complaints are based on patients’ subjective experience. Although reinfections between t_1_ and t_2_ cannot be entirely ruled out, particularly in asymptomatic individuals, no reinfections were reported during the follow-up period. Furthermore, radiological imaging was not possible due to ethical reasons.

## Supplementary Information

Supplementary Material 1.

## Data Availability

The dataset used and/or analysed during this study is not openly available and is available from the corresponding author upon reasonable request.

## Abbreviations

COVID-19: Coronavirus Disease 2019
DLCO: Diffusion Capacity of the Lungs for Carbon Monoxide
FEV_1_: Forced Expiratory Volume in 1 s
FVC: Forced Vital Capacity
LCI: Lung Clearance Index
LLN: Lower Limit of Normal
(Multiplex)-PCR: (Multiplex) Polymerase Chain Reaction
SARS-CoV-2: Severe Acute Respiratory Syndrome Coronavirus 2
ULN: Upper Limit of Normal
