# Supplementary material for: Pulmonary function in non-hospitalized adults and children after mild COVID-19: a single-centre prospective cohort study
Source: BMC Pulm Med. 2025 Nov 6;25:511. doi: 10.1186/s12890-025-04007-y (PMC12593918; doi:10.1186/s12890-025-04007-y)
Supplement: Supplementary file 1 — Supplementary Material 1. [file 12890_2025_4007_MOESM1_ESM.pdf]

Name (Last, First): ________________________________ **Date of birth:** ____.____.___________

Date of SARS-CoV-2 multiplex-PCR: ___.___._______

On which day did you first notice symptoms?

☐ I did not have any symptoms
☐ I do not remember the exact date
☐ Date: ___.___._________

Do you smoke?

☐ No
☐ Yes, _______pack per day for ______ years
☐ I quit smoking in _________ (year), before that I smoked ______ packs per day for ______ years

What was/were the main reason/s for your SARS-CoV-2 test (swab)?

☐ I had symptoms (see below)
☐ Direct contact with a person tested positive
☐ A positive COVID-19 case in my social circle
☐ A positive rapid test (antigen test)
☐ Increased occupational exposure risk
☐ Test required before travel
☐ Test upon return from travel
☐ Screening for private reasons
☐ Screening for occupational reasons
☐ Other: …………………………………………………………

…………………………………………………………

Which symptoms have you experienced?

☐ None

☐ Fever of 38.5°C or higher
☐ Fever, but never exceeding 38.4°C

☐ Cough
☐ Shortness of breath during exertion
☐ Shortness of breath at rest
☐ Rapid breathing / Dyspnoea
☐ Chest pain

☐ Loss of taste
☐ Loss of smell

☐ Sore throat
☐ Runny nose
☐ Ear pain
☐ Skin rash
☐ Muscle pain

☐ Headache
☐ Dizziness
☐ Severe fatigue / exhaustion
☐ Sleep disturbance

☐ Loss of appetite
☐ Weight loss
☐ Abdominal pain
☐ Diarrhea
☐ Vomiting

☐ Joint pain
☐ Conjunctivitis
☐ Swollen lymph nodes
☐ Sweating

☐ Other: …………………………………….

Do you have a dust mite or pollen allergy?

☐ No
☐ Yes, I am allergic to: ………………………………………………………………………………………

………………………………………………………………………………………

Did you experience any of the following conditions as a result of your SARS-CoV-2 infection?

☐ Bronchitis
☐ Pneumonia
☐ Middle ear infection (Otitis media)
☐ Sinusitis (Sinus infection)
☐ none

How long did your symptoms from the SARS-CoV-2 infection last?

☐ I had no symptoms ☐ 0-7 days ☐ longer than 14 days
☐ 8-14 days ☐ I still have symptoms

Did you take any medications because of your SARS-CoV-2 infection that you had not been regularly taking before?

☐ No ☐ Yes, I took the following:
- ☐ A medication for fever / pain: ……………………………………………………………..
- ☐ A decongestant nasal spray / nasal drops
- ☐ Inhalation therapy with salbutamol
- ☐ Inhalation therapy with a corticosteroid
- ☐ Inhalation therapy with another medication: ……………………………………..
  ………
- ☐ An antibiotic
- ☐ Other: ……………………………………………………………………………………………
  ………

Do you feel as healthy as you did before the SARS-CoV-2 infection?

☐ Yes ☐ No, I am still suffering from or have newly developed:

☐ Cough
☐ Chest Pain
☐ Rapid breathing / Dyspnoea
☐ Shortness of breath during exertion
☐ Shortness of breath at rest

☐ Newly developed bronchial asthma
☐ Severely reduced physical capacity
☐ Significantly increased need for sleep

☐ Joint pain
☐ Muscle pain
☐ Swollen lymph nodes
☐ Dizziness
☐ Headache
☐ Sleep disturbance

☐ Loss of appetite
☐ Vomiting
☐ Abdominal pain
☐ Loss of taste
☐ Loss of smell
☐ Others (e.g. see initial symptoms):

☐ Diarrhea
☐ Weight loss
……………………………………………………………
……………………………………………………………

Are you currently taking any medications because of your SARS-CoV-2 infection that you did not regularly take before your infection?

☐ No ☐ Yes, I take the following:
- ☐ A medication for fever / pain: ……………………………………………………………..
- ☐ A decongestant nasal spray / nasal drops
- ☐ A corticosteroid nasal spray
- ☐ Inhalation therapy with salbutamol
- ☐ Inhalation therapy with a corticosteroid
- ☐ Inhalation therapy with another medication: ……………………………………..
  ………
- ☐ Other: ……………………………………………………………………………………………
